# Gene Coexpression Network Comparison via Persistent Homology

**DOI:** 10.1155/2018/7329576

**Published:** 2018-09-19

**Authors:** Ali Nabi Duman, Harun Pirim

**Affiliations:** ^1^Department of Mathematics and Statistics, King Fahd University of Petroleum and Minerals, Dhahran, Saudi Arabia; ^2^Department of Systems Engineering, King Fahd University of Petroleum and Minerals, Dhahran, Saudi Arabia

## Abstract

Persistent homology, a topological data analysis (TDA) method, is applied to microarray data sets. Although there are a few papers referring to TDA methods in microarray analysis, the usage of persistent homology in the comparison of several weighted gene coexpression networks (WGCN) was not employed before to the very best of our knowledge. We calculate the persistent homology of weighted networks constructed from 38 Arabidopsis microarray data sets to test the relevance and the success of this approach in distinguishing the stress factors. We quantify multiscale topological features of each network using persistent homology and apply a hierarchical clustering algorithm to the distance matrix whose entries are pairwise bottleneck distance between the networks. The immunoresponses to different stress factors are distinguishable by our method. The networks of similar immunoresponses are found to be close with respect to bottleneck distance indicating the similar topological features of WGCNs. This computationally efficient technique analyzing networks provides a quick test for advanced studies.

## 1. Introduction

Quantitative skills have become much more essential to distill meaning from the vast emerging and increasing diverse data sets since the technological advances in DNA sequencing that occurred at the end of the 20th century. Modern technological developments in high throughput data technologies such as microarrays and RNA-sequencing enable the generation of terabytes of data in a short amount of time. The type of the data generated comprises levels regarding the abundance of RNA, quantification of protein-protein interactions (PPI), and many other biological molecular interactions. The generated data is embraced for statistical inference and computational analysis including low-level data processing and high-level algorithmic analysis with computations and machine learning techniques. Making use of the data is a reverse engineering approach. Gene coexpression microarrays measure interactive activities of thousands of genes. In network terms, the nodes of the coexpression matrix represent the gene products and the edges of the matrix represent the relationship between the products (usually expressed by correlations). After a chip scanning and an image processing process, a matrix of coexpression values is obtained. The rows of the matrix refer to the gene products while the columns refer to the experiments/samples/tissues. The numeric values of the matrix are the expression values of genes across the experiments. The experiments may be “control versus treated” or “time course.”

The networks constructed based on the gene expression similarity are called gene coexpression networks [1]. They can be named association, correlation, and influence networks [2] as well. Coexpression network analysis requires the selection of a similarity measure between genes and a clustering algorithm to decompose the network into functional clusters/modules following a meaningful experiment design [3, 4]. However, there exist clustering algorithms that do not require a distance matrix as an input, but rather they require the network itself (e.g., some community structure finding algorithms). The modules found by running a clustering algorithm require biological inference.

In order to have a high-level overview of a coexpression network construction and analysis, a few common processes can be summarized [5]:

- Obtaining a filtered data
- Making use of network inference or guilt by association as in clustering
- Enrichment analysis to see the biological relevance of computational outputs
- Extension of the model(s) integrating multiple data types such as mRNA, miRNA data from RNA-req, TF, DNA-binding data from ChIP-seq, and protein interaction data from mass spectrometry

Ideally, network decomposition results in tight clusters/modules with dense intracluster and sparse intercluster connections. Tight clusters are supposed to include biologically relevant genes in terms of functions or residing in the same pathway.

One of the widely used steps in constructing a gene coexpression network is trimming some of the edges based on a threshold [6–8]. Persistent homology, which is first developed to explore the topological features of point cloud data, is a topological invariant, and it addresses the problem of choosing a reasonable threshold. Our method employs persistent homology once the correlation similarity is calculated on the filtered networks.

Persistent homology is a new tool for studying the shape of a point cloud in application areas such as digital images [9, 10], dynamical systems [11], biomolecules [12], and high-dimensional data mining [13]. The persistent homological framework enables us to analyze multiscale networks in a consistent manner [14–16]. The output of the persistent homology of a network can be summarized visually using a *persistent diagram*, and the distance between two persistent diagrams can be measured via *bottleneck distance*. Here, we are using the persistent homology framework to do a threshold-free analysis of weighted gene coexpression networks constructed from 38 Arabidopsis microarray data sets. We list several advantages of our method:

- Our method does not require a choice of fixed threshold as it considers the networks at every possible threshold.
- It gives a more robust result than an analysis of unweighted networks for which the results might depend on the choice of the threshold.
- Persistent diagrams can be used for a standard data analysis method such as cluster analysis.
- Our method eliminates the computational burden of analyzing many networks obtained for different thresholds.

Topological data analysis (TDA) has been applied to biological data before. Arsuaga et al. [17] associate a two-dimensional (2D) point cloud with each array comparative genomic hybridization (aCGH) profile and generate a sequence of simplicial complexes. They use these mathematical objects to identify DNA copy number aberrations by interrogating the topological properties. Camara et al. [18] use TDA for mapping meiotic recombination at fine scales. Comparing to standard linkage-based methods, they find that TDA can deal with a larger number of genomes in a computationally efficient way. Cang et al. [19] propose a support vector machine algorithm for protein classification. They choose the machine learning feature vectors from the persistent homology of the protein structure. Chan et al. [20] use persistent homology to capture both vertical and horizontal evolutions. They show that horizontal evolution exhibits nontrivial topology of dimension greater than zero. Nicolau et al. [21] introduce a topological method that identifies a unique subgroup of estrogen receptor-positive (ER+) breast cancers that express high levels of c-MYB and low levels of innate inflammatory genes. Perea et al. [22] present a novel method based on persistent homology to classify periodic or nonperiodic signals of microarray time series data. Their method successfully identifies the periodic genes in microarray data from the yeast cell cycle.

Here are the main results of the paper:

- We quantify topological features of WGCNs using persistent homology and apply the hierarchical clustering algorithm to the distance matrix whose entries are pairwise bottleneck distance values between the networks.
- The immunoresponses to different stress factors are distinguishable by our method. The networks of similar immunoresponses are found to be close with respect to bottleneck distance indicating the similar topological features of WGCNs. Hence, persistent diagrams of the networks can be used to determine the topological and biological similarities.

## 2. Methods

Topological methods address several problems that arise in biological data analysis [13]. We now summarize three of these which are related to the analysis of our microarray data sets. First of these problems is to extract qualitative information from a given set of data prior to quantitative methods. This might include studying the characteristics of the data space such as determining the connected components, loops, and higher dimensional surfaces. In the biological context, these methods have already been used, for example, in identifying a novel subgroup of a certain disease [17, 21], cataloguing the type of exchange of genomic material [20], classifying protein domain [23], and discovering periodicity in gene expression time series data [22].

The second issue in biological data analysis is the choice of a natural coordinate system. A particular choice of a coordinate system might not have an essential meaning during the analysis. Topological methods, which are coordinate-free and depend only on the chosen metric, enable us to compare the data sets given in different coordinate systems where there is a concept of similarity in general, not only the Euclidean metric.

Thirdly, fixing an optimal parameter in conventional clustering algorithms might not reveal sufficient information about the data set under consideration. Hence, it is preferred to consider the entire set of parameters at once. This raises the question of what the relationship between the information obtained from different parameters is. Topology deals with this problem via the concept of *functoriality* which is used to compute the topological invariants from discrete approximations.

Topology ideally aims to find the homeomorphism type of a topological space. Roughly speaking, we would like to classify the spaces up to stretching and bending but not tearing and gluing. However, in most of the cases, it is very hard to get the homeomorphism type of a space. Hence, we need to consider other invariants: homotopy, homology, cohomology, and so on. In order to find these topological invariants of a data space, we need to construct a combinatorial approximation of the space called *simplicial complex*:

**Definition 1 .**

A *simplicial complex K* consists of a set of objects, *V*(*K*), called *vertices* and a set, *S*(*K*), of finite nonempty subsets of *V*(*K*), called simplices such that (i) any nonempty subset of a simplex is also a simplex, (ii) every one element set {*v*}, where *v* ∈ *V*(*K*), is a simplex, and (iii) the intersection of any two simplices is also a simplex.

The *dimension of a simplex σ* ∈ *S*(*K*) is defined as dim(*σ*) = |*σ*| − 1: simplices consisting of a single element are zero-dimensional, and simplices consisting of two elements are one-dimensional and so on. The *dimension of the complex *dim(*K*) is defined as the largest dimension of any of its simplices.

**Figure 1:**
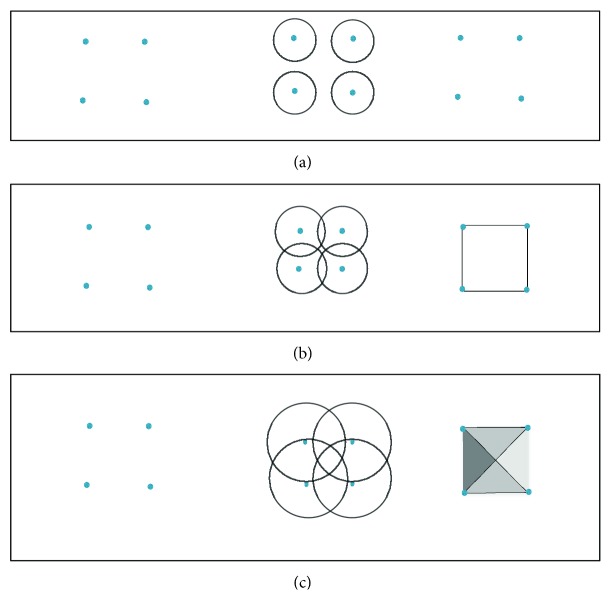
Construction of Rips complex. The leftmost figures show point cloud data, the middle the covering of the balls centered at each point, and the rightmost Rips complex for the corresponding balls of each radius.

One can construct a simplicial complex from a data set in ℝ^*n*^ using a *Rips complex* (see Figure 1).

**Definition 2 .**

Let *X* = {*x*_*α*_} be a set of points in ℝ^*n*^. The *Rips complex R*(*X*, ∈) (also called Vitoris-Rips complex) is the simplicial complex whose *k-*simplices correspond to *k* + 1-tuples of points {*x*_*a*_}_0_^*k*^ which are pairwise within distance ∈.

The Rips complex can be considered a variant of Čech complex construction. The problem with the Čech construction is that it is computationally expensive as it requires the storage of simplices of various dimensions. On the other hand, the Rips complex is computationally more efficient as the edges in the complex completely determine the complex. Although there are other efficient complexes (e.g., witness complex and alpha complex), in this work, it is sufficient for us to use the Rips complex because of the moderate size of the filtered microarray coexpression data under study.

Once we obtain the simplicial complex *K* from our data set, we assign a vector space via homology, which is a classical invariant within algebraic topology. We now summarize the construction of the homology. One can refer to [24] for a more detailed description and properties.

(i) The first step is to construct the chain group *C*_*p*_, which consists of the formal sums of the *p*-dimensional simplices *c* = ∑*γ*_*i*_*σ*_*i*_, where *γ*_*i*_ are in a field *F*. These formal sums are called *p-chains*.
(ii) The next step is to define the *boundary map*, *∂* : *C*_*p*_⟶*C*_*p*−1_ which maps each *p*-chain to the sum of *p* − 1-dimensional subsets, *faces*, of its *p-*simplices. One can easily check that the square of the boundary map is zero; that is, *∂*∘*∂* = 0.
(iii) Hence, one obtains the following *chain complex*:
  (1) $$\begin{array}{c} \ldots ⟶C_{p+1}⟶C_{p}⟶C_{p-1}⟶\cdots \end{array}$$
(iv) As a result of *∂*∘*∂* = 0, one can define the *pth homology group H*_*p*_(*K*) as the quotient of the *p-cycles Z*_*p*_, elements of *C*_*p*_ which are mapped to 0 by *∂*, and *p-boundaries B*_*p*_, which is the image of *C*_*p*+1_ under *∂*.

As the coefficients of the *p*-chains are chosen from a field *F*, the resulting abelian group *H*_*p*_(*K*) is torsion free. Hence, *H*_*p*_(*K*) = *Z*_*p*_/*B*_*p*_ = *F*^*β*_*p*_^ is a vector space over *F*, with rank *β*_*p*_. This number *β*_*p*_ is called *pth Betti number* and is equal to 0 if *p* is greater than the dimension of *K*.

Given a *simplicial map f* : *K*⟶*L* between simplicial complexes *K* and *L*, where *f* takes simplices to simplices and it is linear, and *f* induces a homomorphism on homology *f*_*p*_ : *H*_*p*_(*K*)⟶*H*_*p*_(*L*). This functoriality is crucial, when we next introduce the concept of persistence.

We now review the persistent homology which is the main mathematical tool that we use in our study. One can refer to the survey articles [13–16] for more detailed discussions. Given a fixed possibly obtained from the distribution of a data set *X*, the Rips complex *R*(*X*, ∈) is in most cases not sufficient to extract details about the shape of *X*. Hence, one needs to check *R*(*X*, ∈) for different values to get a satisfactory level of homological information. In order to determine the topological features that persist in a parametrized family of spaces, we construct the family of spaces using the Rips complexes *R*(*X*, ∈_*i*_) of a data set for increasing parameter values {∈_*i*_}_*i*_^*n*^. The inclusion maps give us a filtration of spaces:

(2) $$\begin{array}{c} R\left(X,\in_{1}\right)↪R\left(X,\in_{2}\right)↪\cdots ↪R\left(X,\in_{N}\right). \end{array}$$

We next obtain the following sequence of vector spaces and linear maps using the functoriality of homology:

(3) $$\begin{array}{c} H_{k}\left(R\left(X,\in_{1}\right)\right)⟶H_{k}\left(R\left(X,\in_{2}\right)\right)⟶\cdots ⟶H_{k}\left(R\left(X,\in_{N}\right)\right). \end{array}$$

One decomposes this algebraic structure into summands of the form 0⟶*F*⟶⋯⟶*F*⟶0, which points out the spaces *R*(*X*, ∈_*i*_), where a certain homological feature (i.e., connected components, loops, and voids) manifests. The set of these summands corresponds with finite subsets of *P* = {(∈_birth_, ∈_death_) | ∈_birth_ ∈ [0, ∞), ∈_death_ ∈ [0, ∞), ∈_birth_ < ∈_death_}, where ∈_birth_ and ∈_death_ are the birth and the death times, respectively. The *persistence* of a feature is defined as the difference between its death and its birth time. The features with larger persistence provide the most important information about the topology, while the features with short persistence are most of the time considered noise [19, 20, 23, 25].

The set *P* can visually be represented as a collection of points of the first quadrant of the coordinate plane. This visual representation is called *persistence diagram* and can be considered a replacement for Betti numbers in the case of a collection of thresholds {∈_*i*_}_._ With the help of the persistence diagram, one can identify the significant homological features, which are located far from the main diagonal and filter the noisy features, which can be found near the main diagonal. For example, for *k* = 0, we obtain the persistence of the connected components, and for *k* = 1, we obtain the persistence of the loops.

**Figure 2:**
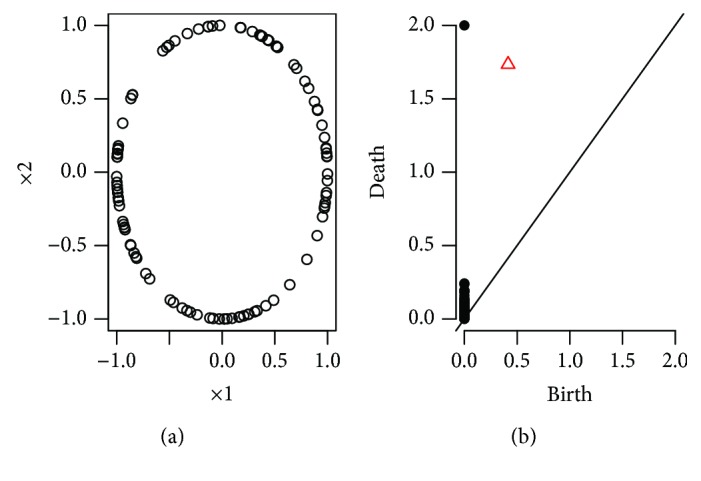
(b) is the persistent diagram of point cloud data on (a).

**Figure 3:**
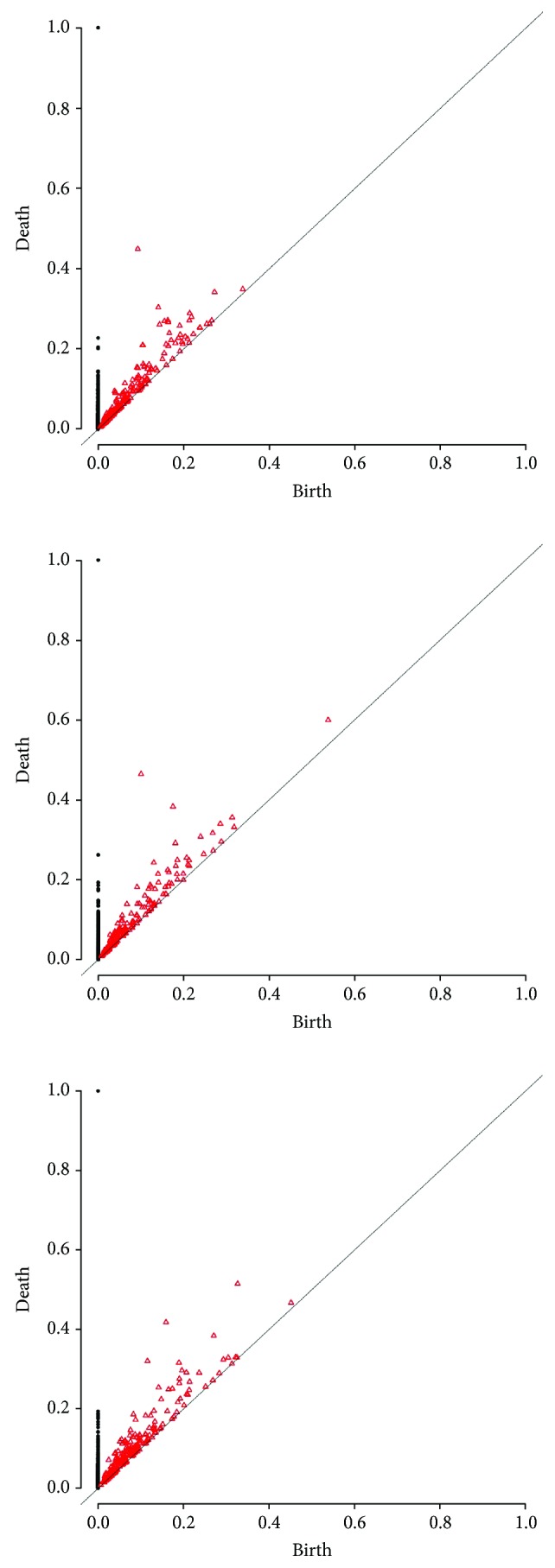
Persistent diagrams of experiments 5, 6, and 8.

**Figure 4:**
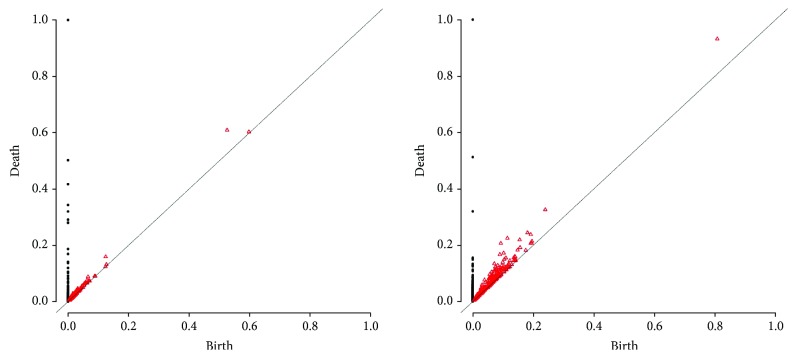
Persistent diagrams of experiments 31 and 32.

In Figure 2, a point cloud data and corresponding persistent diagram are shown. The zero-dimensional features are represented by black dots. The persistent one-dimensional feature represented by a red triangle reveals the existence of the big loop. In this work, we are mainly interested in the evolution of the connected component of the networks. Hence, the birth time of each component is fixed as 0. As the threshold increases, the number of component decreases and no new component is born (Figures 3 and 4).

In order to compare two networks, we need to find the distance between the persistent diagrams of these two networks. The most common metric to measure the distance between two persistence diagrams is the *bottleneck distance* which is known to be stable under small perturbations due to the bottleneck stability theorem [26].

**Definition 3 .**

The *bottleneck* distance between two persistent diagrams PD and PD′ is defined as

(4) $$\begin{array}{c} d_{B}\left(PD,PD^{\prime }\right)=\underset{\lambda }{\inf}\underset{p\in PD}{\sup}{\left\|p-\lambda \left(p\right)\right\|}_{\infty }, \end{array}$$

where *λ* ranges over all bijections from *P* to *P*′ and ‖·‖_∞_ is the *L*^∞^-norm on the plane.

As the diagonals are also considered in the diagram with infinite multiplicity, any bijection *λ* can map an extra point in the diagram with a point on the diagonal. Hence, it is not necessary to have the same number of points in the diagrams. The stability of the bottleneck distance is given by the following inequality:

(5) $$\begin{array}{c} d_{B}\left(PD,PD^{\prime }\right)\leq 2d_{GH}\left(X,Y\right)\leq 2d_{H}\left(X,Y\right), \end{array}$$

where PD and PD′ are persistence diagrams for the data sets *X* and *Y* in a Euclidean space, respectively, and *d*_H_ and *d*_GH_ are Hausdorff and Gromov-Hausdorff distances (Equation (1) in [22]). The bottleneck distance is the *q* = ∞ version of the Wasserstein distance defined as

(6) $$\begin{array}{c} d_{q}\left(PD,PD^{\prime }\right)={\left[\underset{\lambda }{\inf}\;\underset{p\in PD}{\sum }{\left\|p-\lambda \left(p\right)\right\|}_{\infty }^{q}\right]}^{1/q}. \end{array}$$

The Wasserstein distance *d*_*q*_ includes all the differences between the diagrams while the bottleneck distance *d*_*B*_ measures the single largest distance between the persistence diagrams. Thus, we have

(7) $$\begin{array}{c} d_{B}<d_{q}. \end{array}$$

The Wasserstein distance is also more sensitive to the small differences due to noise as the sensitivity of *d*_*q*_ decreases when *q* increases.

The distance between networks can be used to determine the topological and biological similarities. In this paper, we use the bottleneck distance *d*_*B*_ to construct a distance matrix whose entries are the pairwise distance between the experiments represented as networks. The networks are then clustered using Ward's hierarchical clustering algorithm applied in *R*. The optimum number of clusters is predicted using both the Bayesian information criterion (BIC) and the within sum of squares (WSS).

## 3. Results

The pathogen resistance microarrays for *Arabidopsis thaliana* data sets are downloaded from the Gene Expression Omnibus (GEO) database. The data sets can be accessed entering the GEO ID into the query field in the link https://www.ncbi.nlm.nih.gov/geo/query/acc.cgi.  Table 1 summarizes the data sets based on stress groups and stresses. The existing four stress groups: PTI, bacteria, induced resistance, and fungi, belong to 6, 6, 16, and 10 experiments, respectively. The studies with the pathogen *Botrytis cinerea* are the most abundant.

Persistent homology application workflow starts with the robust multiarray average (RMA) normalization for the data sets. R Bioconductor affy package is used for the normalization of Affymetrix data. The two-color microarray data sets are preprocessed using the marray and Agi4x44PreProcess libraries. The expression matrices are constructed for each microarray experiment independently. Each experiment comprises several microarrays.

After the normalization, highly coexpressed genes are retained through a gene filtering procedure so that only the significantly expressed genes are retained in the data matrix. R genefilter package is used, while any other filtering method could be employed. Around 400 genes are remained after filtering each data [21, 27].

The filtered genes are used to construct the weighted coexpression network using the absolute value of the Pearson correlation as the similarity measure, which is commonly used in a gene expression cluster analysis [28]. We use this measure to calculate the dependence between the expression levels of the genes across samples. These similarity measures take values in the same interval [0, 1], where 0 indicates nondependence and 1 indicates total dependence or maximum similarity. Unlike the standard approach in WGCN analyses [29, 30], we do not need to specify a threshold to punish weak correlations and emphasize strong ones because weak correlation is regarded as noise by persistent homology as explained in the previous section.

In the next step, Rips complexes are generated for each weighted network in order to determine the persistent topological features (connected components, loops, voids, etc.). We then calculate the persistent homology of the Rips complexes. In this study, we only consider 0 and 1 dimensional homologies [17, 18, 20, 22]. According to our knowledge, higher dimensional homology has not been used to analyze microarray data. Higher dimensional features such as voids might not have biological significance in weighted gene coexpression networks.

The results for each network are summarized in persistent diagrams. Afterwards, the distance matrix including the distance values between persistent diagrams is obtained employing the bottleneck distance, which is the most commonly used metric to measure the distance between two persistent diagrams [26]. The persistent homology and bottleneck distance are calculated using R TDA package [31].

**Figure 5:**
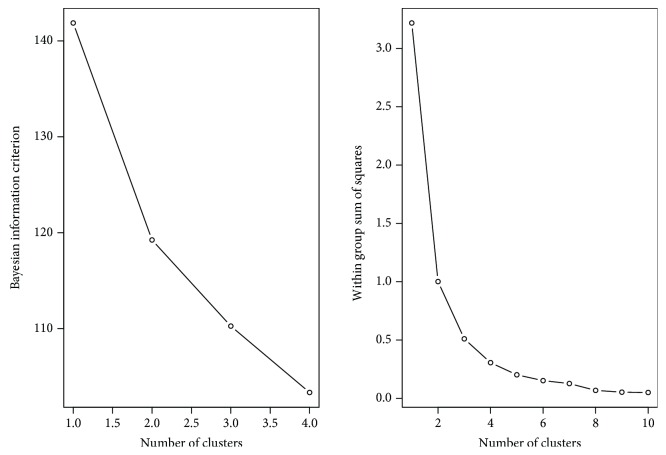
Determination of the number of clusters based on the Bayesian information criterion and the within sum of squares.

**Figure 6:**
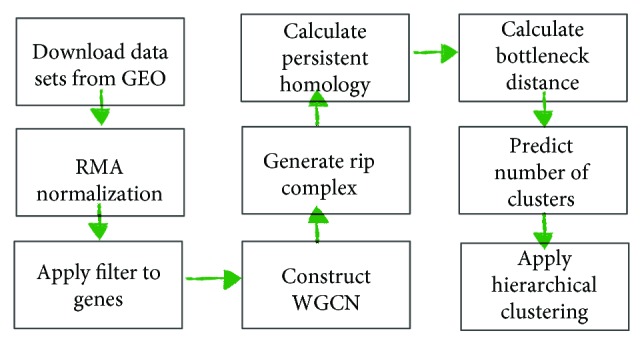
Work flow.

The final distance matrix is used as an input to a hierarchical clustering method in order to compare 38 WGCNs. Hierarchical clustering is frequently used in WGCN analyses since it works well when there are many singleton clusters and when cluster sizes vary greatly [29]. The optimum number of clusters is predicted to be four using both the Bayesian information criterion (BIC) and the *K-means* within the sum of squares method (Figure 5). They are mainly utilized to choose the number of clusters according to the intrinsic complexity present in a particular data set. BIC is employed using the R adegenet package [32]. The workflow is illustrated in Figure 6.

**Figure 7:**
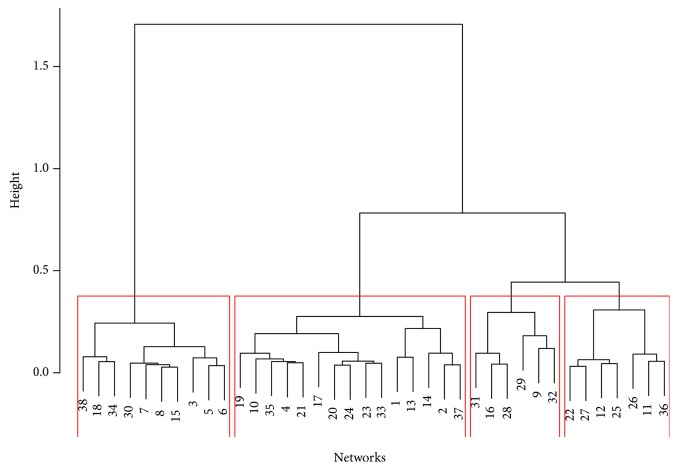
The clustering of zero-dimensional persistent diagrams.

**Figure 8:**
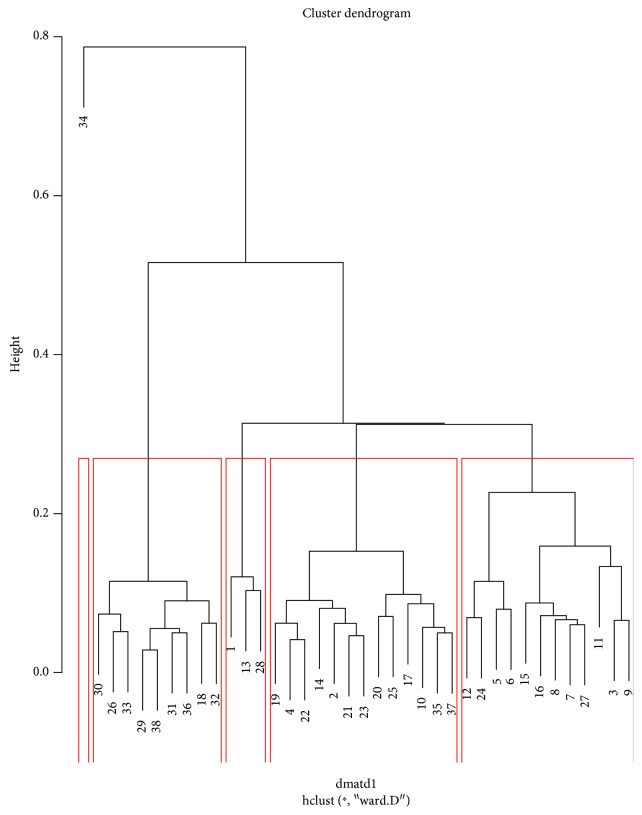
The clustering of one-dimensional persistent diagrams.

The hierarchical clustering using the bottleneck distance with four cluster results in the partition as seen on the dendrogram (Figure 7). The clustering here is obtained from zero dimensional persistent diagrams.  Figure 8 shows hierarchical clustering of one-dimensional persistent diagrams.

We have four different stress groups in our data set: PTI, bacteria, fungi, and induced resistance. The networks from the same stress group were found to be in the same clusters. Here are some examples from the clustering of zero-dimensional persistent diagrams (Figure 7):

- The networks 5, 6, 8, and 18 associated with the stress group PTI are close to each other; hence, they are all in the first cluster. This means that they show similar topological features as a result of the same immune responses to PTI.
- Four out of 6 bacteria experiments (IDs 10, 13, 33, and 35; *Ralstonia solanacearum* and *Pseudomonas syringae*) are located in the second cluster. Thus, we can conclude that the immunity processes of Arabidopsis that are represented in these networks derived from the responses to pathogens *Ralstonia solanacearum* and *Pseudomonas syringae* could share some similarities.
- Five out of 6 experiments in cluster 3 (IDs 9, 28, 29, 31, and 32; *Botrytis cinerea*, *Plasmodiophora brassicae*, and *Fusarium oxysporum* pv. raphani) are related to fungal experiments. Thus, we could say that Arabidopsis has similar immunity responses to *Botrytis cinerea*, *Plasmodiophora brassicae*, and *Fusarium oxysporum* pv. raphani.  Figure 4 shows the persistent diagrams of experiments 31 and 32 of the same study for which the one-dimensional features also resemble each other.
- A similar result can be observed in cluster 4, where 6 out of 7 experiments are identified as induced resistance experiments (IDs 11, 12, 22, 25, 26, and 36; *insecticides*, *SA*, *JA*, and *SR*; *Bradyrhizobium*).
- Networks 19, 20, 21, 23, and 24 from the same study of natural genetic variation for plant disease resistance responses to salicylic acid (SA) are found in the second cluster. The networks 20, 23, and 24 are particularly close to each other revealing similar responses to SA.

Specifically, the similarity between persistent diagrams of networks 5, 6, and 8, which are relatively close with respect to bottleneck distance (Figures 7 and 8), indicates that the coexpression networks of these data sets have similar topological features (see Figure 3). This represents the comparable immunity process in each of these experiments. Indeed, this result agrees with the original study [33]. The goal of these experiments was to identify CPK5ac and CPK11ac early target genes (network 5) and early 22 amino acid peptide of bacterial flagellin (flg22) responsive genes (networks 6 and 8) in Arabidopsis mesophyll cells (networks 5 and 6) and Arabidopsis seedlings (network 8). They discover that CPK5ac and CPK11ac activate the same genes, mimicking flg22.

Consequently, a small bottleneck distance between two persistent diagrams of two networks could have a biological meaning in correspondence with the experiment as some clusters mainly contain specific stress groups. The clustering of the bottleneck distance matrix with the hierarchical algorithm identifies theoretically similar networks based on topological and biological aspects.

On the other hand, some distinct stress groups can have similar WGCNs. For example, induced resistance experiment 16 is in the same group with fungal experiments 9, 28, 29, 31, and 32 of cluster 3. Similarly, PTI experiment 27 is in cluster 4 along with 6 induced resistance experiments (IDs 11, 12, 22, 25, 26, and 36; *insecticides*, *SA*, *JA*, and *SR*; *Bradyrhizobium*).

One can also notice that the topological features of the networks from the same stress group can also be different. For instance, networks 28 and 34 (*Plasmodiophora brassicae* and *Botrytis cinerea*) from the fungal experiments are not close to each other.

## 4. Comparison with the Principal Component Analysis Method

Principal component analysis can also be used to provide a framework to construct and compare weighted gene coexpression networks (WGCNs) employing the same data sets [34]. Unlike in persistent homology, a threshold needs to be determined to construct the network. In this case, the threshold is chosen using mutual information for similarity measurement and a clustering coefficient-based method. PCA is applied to compare WGCNs. Similar WGCNs are projected, that is, clustered, closely on the principal component space using the *K*-means algorithm. Here is the comparison between PCA and persistent homology methods:

- According to PCA results, 6 bacteria experiments on Arabidopsis are associated to different clusters (see Figure 7 in [34]) while 4 out of 6 experiments are located in the same cluster in the persistent homology method. Similar results can be observed for fungi experiments as well. The persistent homology method returns a much better result (i.e., 5 out of 6 fungi experiments are in the same cluster) unlike the PCA approach where fungi groups are spread over distinct clusters.
- According to both methods, PTI experiments 5, 6, 8, and 18 are close to each other (see Figure 6 in [29]).
- Similarly, in both clustering results, induced resistance experiments appear in the same cluster (see Figure 7 in [34]). However, persistence homology is able to detect that networks 20, 23, and 24 revealing similar responses to SA are closer to each other.

## 5. Conclusion

In this paper, a topological data analysis procedure is applied to weighted gene coexpression networks (WGCN) which are constructed from microarray data sets of pathogen-infected *Arabidopsis thaliana*. We quantify topological features of WGCNs using persistent diagrams and apply the hierarchical clustering algorithm to the distance matrix whose entries are pairwise bottleneck distance between the networks. The closeness under the bottleneck distance between the zero-dimensional persistent diagrams of WGCNs indicates similar plant immune responses and conserved signaling pathways. The cluster analysis shows that a small bottleneck distance between two persistent diagrams of two networks could have a biological meaning in correspondence with the experiment as certain clusters dominantly contain specific stress groups.

Our method has recovered what principal component analysis (PCA) approach in the previous studies has identified from the same data set [34]. For certain stress groups, the persistent homology approach outperforms PCA-based clustering.

By using persistent homology along with other methods that complement its strengths and lessen its weaknesses, this method can be used as a tool in WGCN analyses. Indeed, persistent homology can identify the sets of different stress factors from the high-throughput biological data sets such as microarrays. Hence, we provide another evidence that the shape of the data can provide useful information in the study of translational functional genomics.

The proposed method does not utilize any computationally expensive algorithms and can be applied to any similar biological networks such as RNA-seq and protein-protein interaction (PPI) networks.

## Figures and Tables

**Table 1:** Pathogen resistance Arabidopsis microarray data collected. This is the part of the table that appeared in [34], and it is under CC BY 4.0 license.

ID	GEO data set	Plant	Stress group	Stress
1	GSE12856	Arabidopsis	PTI	Nonhost
2	GSE13739	Arabidopsis	Induced resistance	Induced resistance (SA)
3	GSE14961	Arabidopsis	Induced resistance	Induced resistance (SA)
4	GSE15236	Arabidopsis	Fungi	*Fusarium oxysporum*
5	GSE16471	Arabidopsis	PTI	PTI
6	GSE16472	Arabidopsis	PTI	PTI
7	GSE16497	Arabidopsis	Induced resistance	Induced resistance (Aphid)
8	GSE17382	Arabidopsis	PTI	PTI
9	GSE17875	Arabidopsis	Fungi	*Botrytis cinerea*
10	GSE19273	Arabidopsis	Bacteria	*Ralstonia solanacearum*
11	GSE20188	Arabidopsis	Induced resistance	Induced resistance (insecticides)
12	GSE21762	Arabidopsis	Induced resistance	Induced resistance (JA)
13	GSE21920	Arabidopsis	Bacteria	*Pseudomonas syringae*
14	GSE26679	Arabidopsis	Fungi	Golovinomyces cichoracearum
15	GSE26973	Arabidopsis	Induced resistance	Induced resistance (exudates)
16	GSE28800	Arabidopsis	Induced resistance	Induced resistance (chemistry)
17	GSE431	Arabidopsis	Fungi	*Erysiphe cichoracearum*
18	GSE5513	Arabidopsis	Induced resistance	Induced resistance (PTI)
19	GSE5752	Arabidopsis	Induced resistance	Induced resistance (SA)
20	GSE5753	Arabidopsis	Induced resistance	Induced resistance (SA)
21	GSE5754	Arabidopsis	Induced resistance	Induced resistance (SA)
22	GSE5755	Arabidopsis	Induced resistance	Induced resistance (SA)
23	GSE5756	Arabidopsis	Induced resistance	Induced resistance (SA)
24	GSE5757	Arabidopsis	Induced resistance	Induced resistance (SA)
25	GSE5758	Arabidopsis	Induced resistance	Induced resistance (SA)
26	GSE6831	Arabidopsis	Induced resistance	SAR(JA)
27	GSE8319	Arabidopsis	PTI	PTI
28	GSE10426	Arabidopsis	Fungi	*Plasmodiophora brassicae*
29	GSE10713	Arabidopsis	Fungi	*Fusarium oxysporum* pv. raphani
30	GSE13390	Arabidopsis	Bacteria	*Pseudomonas syringae* pv. tomato
31	GSE15880	Arabidopsis	Fungi	*Botrytis cinerea*
32	GSE15881	Arabidopsis	Fungi	*Botrytis cinerea*
33	GSE18757	Arabidopsis	Bacteria	*Ralstonia solanacearum*
34	GSE25838	Arabidopsis	Fungi	*Botrytis cinerea*
35	GSE34081	Arabidopsis	Bacteria	*Pseudomonas syringae* pv. tomato
36	GSE7990	Arabidopsis	Induced resistance	Induced resistance (ISR, *Bradyrhizobium*)
37	GSE8877	Arabidopsis	Fungi	*Plasmodiophora brassicae*
38	GSE31230	Arabidopsis	Bacteria	*Ralstonia solanacearum*

## Data Availability

The microarray data used to support the findings of this study is publicly available in the Gene Expression Omnibus (GEO) database (https://www.ncbi.nlm.nih.gov/geo/).
